# Ingalls on Scarlatina

**Published:** 1840-01

**Authors:** 


Art. V.—*On Scarlatina*, in a Letter addressed to his Son.—By William Ingalls, M.D., &c. &c. Second edition.

Dr. Ingalls details three cases of Scarlatina, in his letter, with the mode of treatment which, during a long experience, he has found most successful. Remarking on the various remedies employed, we were surprised to find Dr. I. declaring that, in the treatment of Scarlatina, he was opposed to the detraction of blood by the lancet or leeches. We have been inclined to regard bleeding as one of the most potent means of subduing this formidable complaint—as indispensable in the highly inflammatory grades of the disease. Dr. I. remarks: " In the whole course of my practice in Scarlatina, *I have never employed blood-letting*, either general or local; and I do not recollect a single instance, in which I have had reason to regret the omission." He adds—"It may be proper to state, my practice has been chiefly within the city of Boston. As the situation of a place, and of course, its climate and soil, the customs and manners of its inhabitants, may have great influence in varying the type of acute diseases, a difference in the mode of treatment may not only be proper, but required." Ptyalism occurred in two of his cases, which could not be traced to the action of mercury. He remarks:

"The ptyalism began to decline in about six days; to *me*, in this case, the cause of the complaint is unknown. Cases of idiopathic ptyalism have occasionally come uder my care from the commencement of my practice; some having all the characters of a mercurial salivation. This circumstance has led me to conjecture it may have been the result of mercury administered at a former period, and having lain dormant, until some change in the system have had the effect of bringing into action its properties of ulcerating the gums and mucous membrane of the cheeks and tongue, and consequently increasing the secretion of saliva. On this subject, however, I have formed no definite opinion.

"In this case of your sister, and also in that of your brother, an account of which follows it, I would remark, the ptyalism was not attended with the swelling and ulceration of the tongue and gums, nor the offensive fœtor peculiar to mercurial salivation."

